# Evaluating gold standard corpora against gene/protein tagging solutions and lexical resources

**DOI:** 10.1186/2041-1480-4-28

**Published:** 2013-10-11

**Authors:** Dietrich Rebholz-Schuhmann, Senay Kafkas, Jee-Hyub Kim, Chen Li, Antonio Jimeno Yepes, Robert Hoehndorf, Rolf Backofen, Ian Lewin

**Affiliations:** 1 Institute of Computational Linguistics, University of Zürich, CH-8050 Zürich, Switzerland; 2 European Bioinformatics Institute, Wellcome Trust Genome Campus, Hinxton, Cambridge, CB10 1SD, UK; 3 NICTA Victoria Research Laboratory, The University of Melbourne, Wilson Ave, Melbourne VIC 3010, Australia; 4 Department of Computer Science, Aberystwyth University, Llandinam Building, Aberystwyth, Ceredigion, Wales SY23 3DB, UK; 5, Ludwigs-University Freiburg, Fahnenbergplatz, D-79085 Freiburg, Germany; 6 Linguamatics Ltd, 324 Cambridge Science Park, Milton Road, Cambridge CB4 0WG, UK

## Abstract

**Motivation:**

The identification of protein and gene names (PGNs) from the scientific literature requires semantic resources: Terminological and lexical resources deliver the term candidates into PGN tagging solutions and the gold standard corpora (GSC) train them to identify term parameters and contextual features. Ideally all three resources, i.e. corpora, lexica and taggers, cover the same domain knowledge, and thus support identification of the same types of PGNs and cover all of them. Unfortunately, none of the three serves as a predominant standard and for this reason it is worth exploring, how these three resources comply with each other. We systematically compare different PGN taggers against publicly available corpora and analyze the impact of the included lexical resource in their performance. In particular, we determine the performance gains through false positive filtering, which contributes to the disambiguation of identified PGNs.

**Results:**

In general, machine learning approaches (*ML-Tag*) for PGN tagging show higher F1-measure performance against the BioCreative-II and Jnlpba GSCs (exact matching), whereas the lexicon based approaches (*LexTag*) in combination with disambiguation methods show better results on FsuPrge and PennBio. The *ML-Tag* solutions balance precision and recall, whereas the *LexTag* solutions have different precision and recall profiles at the same F1-measure across all corpora. Higher recall is achieved with larger lexical resources, which also introduce more noise (false positive results). The *ML-Tag* solutions certainly perform best, if the test corpus is from the same GSC as the training corpus. As expected, the false negative errors characterize the test corpora and – on the other hand – the profiles of the false positive mistakes characterize the tagging solutions. *Lex-Tag* solutions that are based on a large terminological resource in combination with false positive filtering produce better results, which, in addition, provide concept identifiers from a knowledge source in contrast to *ML-Tag* solutions.

**Conclusion:**

The standard *ML-Tag* solutions achieve high performance, but not across all corpora, and thus should be trained using several different corpora to reduce possible biases. The *LexTag* solutions have different profiles for their precision and recall performance, but with similar F1-measure. This result is surprising and suggests that they cover a portion of the most common naming standards, but cope differently with the term variability across the corpora. The false positive filtering applied to *LexTag* solutions does improve the results by increasing their precision without compromising significantly their recall. The harmonisation of the annotation schemes in combination with standardized lexical resources in the tagging solutions will enable their comparability and will pave the way for a shared standard.

## Introduction

The integration of the biomedical literature into the infrastructure of other biomedical data resources requires the identification of entities and concepts, such as proteins and gene named entities [1,2]. Furthermore, the entities have to be linked to specific database entries to achieve interoperability between all data resources (called “grounding” or “normalisation”) [3-5]. Once genes and other entities have been correctly identified, the different data resources and information types can be explored in concerto: Genes can be attributed with known facts such as associated diseases or novel drug candidates [6,7].

### “Which gene did you mean?”

Biologically, a gene is given by one or several genetic sequences that reside on one or several locations on the DNA (genetic loci). According to semantic formalisms a gene is a disposition which is described by the given genetic sequence. By contrast, a protein, a gene product, is a class of molecules with specific properties: composed of amino acids, having a structure and specific functions. A gene can be analysed by sequencing, but the observation of a protein and its functions requires more complex experimental setups. Eventually, observations about proteins (and genes) are kept in reference databases, like UniProt [8] for proteins or the NCBI Gene database [9] (formerly known as Entrez Gene), which also contribute with unique identifiers for grounding or normalisation [10].

Scientists frequently do not distinguish between the mention of a gene or a protein in their publications, since the gene is – in general – transcribed and translated into a protein anyways, i.e. the gene produces multiple instances of this particular protein type, which then exposes their function [11]. In principle, we should have for every protein type a unique database entry which contains the reference information such as a unique identifier, the protein properties and also the names (“labels”) that would help to recognize the protein type in text, called protein and gene names (PGNs). Unfortunately, the existing resources are not quite as comprehensive yet.

More than 500,000 database entities are available that refer to protein types, which also contribute with several million names, synonyms, acronyms, hypernyms and morphological term variants [12]. These names certainly comply with established nomenclature guidelines, but the reporting in the scientific literature is not similarly standardised and produces new term variants on a daily basis [13-15].

### Standards for protein and gene names

There are several “standard” approaches to validate a PGN identified in text. A first approach is to consult the naming guidelines [16-18], to discover that the name (1) may represent a recognized macromolecule (e.g., “hemoglobulin”, “prolactin”), (2) may state the function of the gene or protein (e.g., “methyl-transferase”), (3) may state part of the structure of a protein (e.g., “Cytochrome c oxidase subunit 2”), (4) may indicate that the protein is part of a process (e.g., “Mitochondrial fission process protein 1”), (5) may indicate its action on a target (e.g., “DNA gyrase inhibitor”), (6) may induce a phenotype (e.g., “protein hunchback”), (7) may express chemical or physical properties (e.g., “37.8 kD protein”), or (8) may just be similar to another known protein or gene (e.g., “Myc homolog protein”).

A second and simpler approach, which is another standard, would be looking up the name in a reference database, where the PGN may be linked to a single entry (“unique name”), to several data entries (“polysemous term”), or to no entry at all (“unknown PGN”). A third approach would imply using a PGN tagger, e.g. “Banner” [19] a state of the art machine learning based tagger, and any finding of a PGN would be considered a true positive ignoring for the moment missed terms (false negatives, FN) and the wrongly identified terms (false positives, FP). Other tagging solutions have been suggested and tested as well, but may be used to a lesser degree [20]. A preliminary comparison of *ML-Tag* solutions has already been performed [21], but was executed to judge methods for the identification of protein interactions from the scientific literature, and as a consequence did not cover the same scope as this analysis.

As a last approach, the different GSCs could be consulted, which have been produced to denote the correct mention of a PGN in the scientific literature. The following GSCs have to be considered (cf. Table 1): (1) Jnlpba [22] (2004) which stems from the Genia corpus, (2) BioCreative-II [23] (BC2, 2007) for human PGNs, (3) PennBio corpus [24] (2006–2007) about oncology, and (4) FsuPrge corpus [25] (2009) on gene-regulatory events. The CRAFT corpus [26] has not been considered in this study, since it covers full text articles and was not yet available during the experimental phase of this work.

**Table 1:** A number of gold standard corpora have been delivered to the public for the evaluation of PGN tagging solutions

**Name**	**Release**	**# Annot.**	**# Units**	**Topic**
Jnlpba	2004	6,142	401 abs.	Subset of Genia
BioCreative-II	2005	5,144	4,171 sent.	Human proteins
PennBio	2006–07	18,148	1,414 abs.	Oncology
FsuPrge	2009	59,483	3,236 abs.	Gene regulatory processes

Considering the different solutions to represent the same or similar standards, the question arises, how these different solutions relate to each other. Can we characterize, which portion of the semantics representation they share and where they differ? Although Banner is only one piece in the puzzle, it can be used as the starting point: first as a means to judge the compliance of the GSCs and second, as a means to compare against other PGN tagging solutions measured across all corpora.

Note that all evaluations are concerned with the surface forms of PGNs in text and in the other semantic resources, thus our analysis is focused to the identification of gene mentions. From a different angle, the presented study still allows to derive, whether or not a PGN mention given by a tagging solution is linked to a concept identifier (see Table 2), which is deemed more precious than the pure identification of a PGN.

**Table 2:** The PGN tagging solutions are incorporate different components, i.e. lexical resources or trained machine-learning based entity recognizers

**Tagger**	**Acronym**	**Tagger**	**Lexical**	**# Lexical**	**Id**	**Training**	**FP**
**name**		**type**	**resource**	**entries**		**data**	**filter**
Banner		ML	–	–	No	BC2	Banner
Chang2	**Ch2**	ML	–	–	No	BC2	Chang2
Abner (BC1)		ML	–	–	No	BC1	Abner (BC1)
Abner (Jnlpba)		ML	–	–	No	Jnlpba	Abner (Jnlpba)
SwissProt	**SP**	Lex	SwissProt	228,893	Yes	–	BNC
SwissProt (GP7)		Lex	GP7	868,050	Yes	–	BNC
BioLexicon		Lex	BioLexicon	653,212	Yes	–	BNC
GeneProt 7.0	**GP7**	Lex	GP7	1,725,500	Yes	–	BNC
Wh-Ukpmc		Lex+ML	SwissProt	228,893	Yes	–	BNC, Chang2
Wh-Ukpmc (GP7)	**WH7**	Lex+ML	GP7	868,050	Yes	–	BNC, Chang2
Gnat (human)		Lex+ML	Human genes		Yes	BC2	–
Gnat (all)		Lex+ML	11 species	80,000	Yes	BC2	–
Gnat-GN (all)		Lex+ML	11 species	80,000	Yes	BC2	–

### Is Banner the best tagging solution?

In the search of the best tagging solution, a number of hypotheses and test scenarios can be evaluated. State of the art PGN tagging solutions like Banner have not fully solved the PGN tagging task yet, the question remains whether it is possible to introduce a solution that performs better than Banner. It is easy to derive that Banner will certainly perform best on the BioCreative II corpus, which has been used to train the tagging solution, but how big is the performance loss, when we evaluate it on other GSCs?

It is also possible to evaluate PGN tagging solutions that have been trained on other GSC, e.g. Abner [20] (BioCreative-I) and Abner (Jnlpba), and compare them against Banner, leading to the obvious assumption that each solution may achieve better performance on the test corpus of the GSC they have been trained on. But which PGN tagger will perform best across all GSCs? The answer to this question requires that all PGN tagging solutions are tested against all GSCs.

It is also possible to explore PGN tagging solutions that have not been trained on any of the GSCs, e.g. dictionary lookup based solutions from the Whatizit [27] infrastructure. Several taggers are available that can be distinguished according to the lexical resources that have been incorporated into the annotation solution. Two questions arise: Which one performs best and on which GSC, and is it possible to correlate the tagging performance with the type of lexical resource that has been incorporated?

Another observation results from the previous considerations. If we test a selection of PGN tagging solutions across different GSCs, then we will find out that the taggers miss annotations (FNs) that are specific for one GSC in contrast to another, and that some tagging solutions are more alike in the production of unconfirmed annotations (FPs) than others. It becomes clear, that the PGN taggers may have similar FP profiles and a given GSC may induce similar FN profiles. Are these profiles giving us better insights, which setup for a PGN tagging solution is required to achieve the best performance on a given corpus or are selected corpora even more characteristic for a given set of PGNs after all? Even beyond this result, can we better analyse what distribution of PGN representations we would have to expect in the scientific literature in general?

It becomes clear that the comparison of as many PGN tagging solutions against as many GSCs as available leads to a matrix where each solution shows its best fit to a GSC and eventually, it becomes possible to reverse engineer which resources induce a PGN tagging solution that produces the best results against the given GSC.

### How to evaluate a PGN mention?

A priori, it is only necessary to apply all PGN tagging solutions against all corpora, count the true positives, false positives and false negatives and finally calculate precision, recall and F1-measure. Any GSC – in its own rights – would claim this result as the only correct solution. Unfortunately, as it is often the case, a more complex solution is required to produce results that are independent from a single corpus.

Ideally, we would like to measure the correct assignment of an identifier to the PGN mention in the text, where the identifier points to an entry in the reference database [10]. Since PGN mentions are often polysemous and have been reused in a number of database entries, it is impossible to derive automatically the identifier which should be the preferred choice for the author. A recent experiment has also shown that authors as well fail to use database entries – even when supported by experts for doing so – and rather prefer to use terminology only when specifying PGNs in theirs manuscripts [28-30]. It is in the same way impossible to make a clear distinction between gene mentions and protein mentions, and name ambiguities between genes and proteins, e.g. 'hypA’ versus 'HypA’, may result from the annotation guidelines [11].

The term boundaries are certainly crucial for the characterization of the term and its semantics. Again, it has been claimed that the difficulties in determining the correct term boundaries do result from the complexity of the naming guidelines, which – in this case – have been used for the production of a GSC [11]. As an example, the organism names preceding a protein name may or may not be part of the protein name. The authors address the problems by defining the tagging of proteins as the prime target of the extraction task, and also distinguish the protein tags and the long-form tags as two annotation types, where the latter is formed by optionally extending the boundaries of the protein tag whenever the name boundaries are difficult to determine.

In essence, exact boundary matching gives clear evaluation guidelines, but will ignore term variability, i.e. ignores alternative annotations by a PGN tagging solution that does cope with term variability. When relaxing the boundary criteria, either by treating morphological term variability as a single entry (e.g. “HZF-7” vs. “HZF-7 protein”) or by the use of flexible matching [31] (e.g. cosine similarity, nestedness) or by allowing alternative term mentions (see BioCreative II, Alternative Gene List) the performance of the tagging solutions increases, but the precise interpretation of the results is reduced.

### Background

The following section gives an overview on different solutions for PGN tagging. In principle, a classifier can be trained on a GSC and then correctly identifies the PGN boundaries (“mention identification”), but would not attribute a database identifier to the PGN mention. The latter requires at least the use of one or more lexical resources.

#### ***PGN tagging solutions***

Yamamoto distinguishes three different basic approaches for the identification of bio-entities: (a) exact and approximate string matching of entities [32,33], (b) handcrafted rule-based approaches [34,35], and (c) machine learning solutions [36,37]. In addition, hybrid methods exist that integrate dictionaries with machine-learning approaches [38-40].

In principle all gene taggers fulfill a number of specific tasks. In the first place they identify a stretch of text that appears to represent a gene [41]. This judgment requires that the morphological and syntactical structure of the gene is identified from text [42,43]. The second step is the use of the contextual information to achieve term disambiguation or normalization of the named entity to a concept identifier [44,45]. Both results can be achieved with a single processing step, for example in the use of dictionary based methods, but under-perform in terms of context-sensitive disambiguation of terms and entities in comparison to the ML approaches.

One solution for the gene identification is the use of a terminological resource that covers the full set of PGNs [45]. Such resources have been generated from public scientific databases, e.g. from UniProtKb, or from a set of public resources in combination or can also be produced from the scientific literature [46-48]. The size of the terminological resources is crucial for the identification task, since only a comprehensive resource can ensure that all entities are identified [49]. On the other hand, a larger terminological resource increases the noise ratio of the gene tagger, since less common and ambiguous gene names raise the false positive rate. Last but not least, the use of existing terminological resources leads to the advantage that the gene can be linked to their database resource [15,44,50,51], which provides further relevant information about the gene.

As mentioned before, typical machine-learning solutions solve the mention identification task and have been trained and tested on a GSC [19,52,53]. Hybrid methods require the integration of a dictionary and a machine-learning approach [38-40].

#### ***Normalising gene entities***

The PGN tagging solutions provide one or several types of normalisation. The first type of normalisation only affects the mention in text. The PGN tagger could provide a left-most or right-most longest match to a PGN or could just instantiate the normalisation according to a GSC (“mention normalisation”). In the second type of normalisation, the PGN tagger would ensure that the mention exists in a terminological resource (“lexical lookup normalisation”, see Table 3), where the match may or may not consider any morphological variability (HZF 7 vs. HZF1-7 vs. HZF/7) or other modifications to the term.

**Table 3:** All available terminological resources have been compared against each other to identify the most comprehensive and the most universal ones

			**Tagger**							
**Match**	**Corpus**	**# Entries**	**SwissProt**	**[%]**	**Biolexicon**	**[%]**	**SwissProt (GP7)**	**[%]**	**GP7**	**[%]**
	SwissProt	228,893			208,069	90.9%	121,369	53.0%	135,018	60.0%
Exact	BioLexicon	653,212	207,976	31.8%			243,573	37.3%	422,477	64.7%
	SP(GP7)	868,050	121,030	13.9%	243,271	28.0%			860,094	**99.1%**
	GP7	1,725,500	134,275	7.8%	421,520	24.4%	859,536	49.8%		
	SwissProt	228,893			213,009	93.0%	201,633	88.1%	206,047	90.0%
Alias	BioLexicon	653,212	229,759	35.2%			375,550	57.5%	585,205	89.6%
	SP(GP7)	868,050	219,185	25.3%	364,171	42.0%			865,590	**99.7%**
	GP7	1,725,500	267,947	15.5%	644,115	37.3%	956,314	55.4%		

The dictionary-based tagging solutions make use of different terminological resources and the lexical resources have been compared as described here (Table 3). One lexical resource has been used as a component in a tagging solution (called “tagger” in the in Table 3) and has been applied to the lexical resource to annotate the content (called “corpus”). Two different methods for the matching have been applied: (1) exact matching (upper part of the table), and (2) matching with morphological variability (“alias” matching, lower part). The cross-tagging shows the intersections between the different resources and overall it becomes clear, which terminological resource comprises fully or partially one of the other resources.

The third type of normalisation, which is the most advanced one, would select the most appropriate reference to a database entry ("concept normalisation"), but would not allow mulitple references to database entries.

Different approaches have been proposed to normalize the entities, but no complete solution has been presented yet. Approaches that anticipate normalization are more restrictive in contrast to other PGN taggers, since they attempt to narrow the selection down to the most specific assignment amongst all candidates. Normalisation approaches include the following solutions: (1) delivery of all the entities that could be resolved to the PGN, (2) selection of a species that is automatically used for the normalization independently from the mention of any other species in the text, (3) selection of the species from the text that could indicate the right resolution, and (4) use of genomic information and database information for the gene normalization [44,51,54-57]. A toolkit for the normalisation is available from [58] and the evaluation has been addressed as part of the BioCreative III challenge [59].

Combining different identification approaches for genes leads to a modular setup that enables shaping the outcomes to the demands of the identification system. Both, high recall versus high precision can be achieved through different system setups [45]. Ultimately, the tagging solutions have to comply with the lexical resources, with the coding standards and with the GSCs for benchmarking measurements to demonstrate the best performances.

### Overview

In this study, we evaluate and compare the performance of several PGN tagging solutions against available PGN gold standard corpora. Then, we analyse which features of specific PGN taggers lead to a drop in their performance, and what characteristics of the GSCs influence their performances. Furthermore, we analyse the size of the lexical resources and the impact in their performance, and to which extend false positive filtering improves the results. Finally, we compare the different tagging solutions and perform error analysis. To do so, we generate false positive and false negative profiles for each tagging experiment and compare the profiles across tagging solutions and corpora to describe the error profile that is characteristic for each tagger-corpus pair.

## Materials and methods

In this section, we present the GSCs and annotation solutions used in this work. The corpora and annotation solutions have been standardized to a common format (IeXML) [60], which allows an easier integration of the different components of the experiments. The evaluation tool used in this work has been developed and extensively tested as part of the CALBC project^a^.

### Gold standard corpora

Gold standard corpora have different profiles, e.g. size, topic, release date, and annotation guidelines that will impact the performance of the tagging solutions. Table 1 contains statistics of the different selected corpora.

The Jnlpba corpus is based on the Genia corpus [22], which contains annotation for different entity types linked to molecular biology such as PGN, cell type, and cell line, all being compliant with the Genia ontology.

The BioCreative-II corpus covers human genes and proteins and contains sentences instead of complete Medline^®^ abstracts [61]. It makes use of an alternative gene-list in addition to the regular list of genes for example, the GL (= GeneList) contains (1) P00027967A0207 | 11 31 | secretory HIantibodies, while the AGL (= Alt Gene List) also includes: (2) P00027967A0207 | 11 21 | secretory HI, (3) P00027967A0207 | 20 21 | HI, and (4) P00027967A0207 | 20 31 | HI antibodiesfor the same annotation example.

The Medline abstracts in PennBio have been developed with a focus on oncology [24]. The FsuPrge corpus is the largest of all and has a focus on molecular mechanisms such as gene regulation.

### Tagging solutions

We have tested several tagging solutions, with different underlying approaches. First, we have used state of the art *ML-Tag* approaches for gene mention identification. Second, we have used standard *LexTag* solutions where different terminological resources for PGNs have been integrated with and without disambiguation techniques. Last, we have combined the first and the second type to filter out false positives after dictionary lookup.

The *ML-Tag* approaches comprised Abner (BC1) trained on BioCreative-I, Abner (Jnlpba) trained on Jnlpba, and two taggers trained on BioCreative-II (Banner, “Chang2”) based on Conditional Random Fields (CRF) [20,62]. The Banner tagger has been downloaded from the distribution site^b^. The Chang2 tagger is a CRF model trained on BioCreative-II data with a set of features as given in [62]: (1) all character n-grams of length 2 to 4, (2) inclusion of capitalization (token starts or ends with a Capital letter, or Capital letters only etc.), (3) length of the tokens (one character, two characters, between three and five), (4) inclusion of digits (1 digit, 2 digits, only digits), and (5) contained punctuation symbol or a Greek character. In addition a contextual window of plus or minus 2 tokens is employed (called “offset conjunctions”). The model is implemented as CRF (using mallet).

For the *LexTag* solutions, we tested publicly available Whatizit modules from UKPMC and other research (cf. Table 2) [27,63]. In addition, we have compared the two latest versions of the Biothesaurus (version 6.0 and 7.0, GP6 and GP7, respectively) as part of the existing solutions to identify the importance of the lexical resource.

SwissProt (SP) is the Whatizit-SwissProt module integrated into different text mining solutions such as EBIMed [64] and PCorral [65]. It uses terms retrieved from the SwissProt subset of UniProtKb obtained in 2007. SP(GP7) is the updated version, which comprises the full selection of SwissProt PGNs from Biothesaurus 7.0. The tagging of genes applies morphological variability to terms, i.e. accepting separators ([- /]), initial capitalization, and singular-plural variability (“alias matching”), since this morphological variability is very common in the use of gene names [66]. This approach is similar to approximate string matching, e.g. Levenstein distance, but is more specific and thus better suitable for the comparison of PGN terms. Alternative methods include the automatic generation of a large dictionary resource containing all terms exposing the same term variability and then apply exact matching [42].

Finally, all tagged PGNs will be removed that are too unspecific, i.e. those terms that are part of the general English language and would be difficult to attribute to a specific gene or protein. This approach applies to all PGNs that appear in the British National Corpus (BNC)^c^ with a frequency rate that is higher than the one of “insulin” (“basic disambiguation” or “BNC disambiguation”). It has been used in all *LexTag* solutions.

The GP7 solution makes use of the full content from Biothesaurus 7.0, applies morphological variability and basic BNC disambiguation only. Biolexicon integrates the full BioLexicon content into the same approach. The two solutions Wh-Ukpmc and Wh-Ukpmc (GP7) implement the same solution as before with Biothesaurus 6.0 and 7.0, respectively, and in addition we apply FP filtering with the Chang2 tagger after the basic BNC disambiguation to increase precision (“false positive filtering”).

The Table 3 gives an overview on the content used for building the dictionary-based solutions and demonstrates how the lexical resources differ in their size and content. The comparison makes use of exact and alias matching (see above) where a dictionary is incorporated into a lexical tagger (see above) for the matching of terms in the other lexical resource. According to our manual evaluation, GP7 in contrast to GP6 exposes reduced term variability across the resource to be more compliant with the naming standards in the biomedical research community.

**Figure 1:**
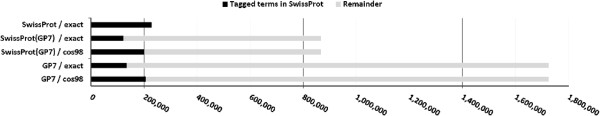
**The tagging solutions have been used to annotated the lexical resource to determine how they comply with naming standards.** The figure shows the tagging of entries from larger dictionary resources (SwissProt (GP7), GP7) against the smaller lexical resource (SwissProt). When using exact matching, only about 53% and 60% of the terms in the smaller resource can be identified by SwissProt (GP7) and GP7, respectively; whereas 88% and 90%, respectively, have been tagged when using alias matching. The same results are produced when using the small lexical resource as tagger against the larger lexical resources. The first experiment using exact matching demonstrates that the larger lexical resource uses slightly different notation standards which can be ignored when using alias matching. It is also remarkable that the smaller lexical resource seems to be already very effective in the tagging of the genes in the corpora indicating that the core terminology for gene mentions is already included in a comprehensive way.

In Figure 1 the small terminological resource generated from the SwissProt part of UniProt, which only contains 228,893 terms, has been compared against two selections from GP7, which is the version 7.0 release of the BioThesaurus forming the largest lexical resource for gene names. The figure visualizes the portion of SP that can be matched by GP7 terms in exact and alias matching and shows how the two lexical resources differ in their naming standards, how this difference disappears under alias matching and that – in the latter case – almost all terms from SP are covered by GP7. This comparison shows that the corpora match a significant portion of core terms that are already included in the SP lexical resource.

Finally, the Gnat gene mention tagger and the gene normalization solution have been tested against the corpora [42]. The Gnat gene normalisation mode has been used, since the gene mention mode of Gnat is based on Banner. The tagger has been applied in different modes using only the dictionary for human genes (GNAT/hum.) or for all species (GNAT/all).

In the latter case, the full set comprises 11 dictionaries for human and the most common model organisms (in total 80,000 entries).

### Evaluation

For all tagging experiments against the corpora, we have calculated Precision, Recall and F-measure defined as: Precision $P=\frac{\left|X_{I}\cap X_{M}\right|}{X_{I}}$, and Recall $R=\frac{\left|X_{I}\cap X_{M}\right|}{X_{M}}$, where *X*_*I*_ is all entities identified by the PGN taggers and *X*_*M*_ is all entities which are (manually) annotated in the GSC. The F1-measure has been calculated as $\frac{2\text{PR}}{P+R}$.

In addition, and in order to obtain a better understanding of the similarities and differences of the taggers, we have collected and counted the FP and FN results separately, sorted them according to their frequency and selected the most frequent errors for our analysis (*N*=100, (Additional file 1): Tables S10, S11, S12 and S13). The FP results from all experiments have been normalized to produce the representation of the FP profile, i.e. the FP results from each corpus formed a single entry in the normalized vector. The FP profiles were populated with the FP error frequency and have then been used to measure the correlation between the error profiles. The FN profiles were produced in the same way using the FP error frequencies. The same profiles were exploited for the similarity comparisons as well as clustering of the results (using R × 64 2.1.4.0), based on a hierarchical clustering where at each iteration step the distances between the clusters is determined by the Lance-Williams dissimilarity update formula and by the complete linkage method that supports identification of similar clusters^d^.

To calculate precision, recall and F1-measure, the evaluation of the annotated documents against the corpora has been performed with exact matching, but as well with a more relaxed matching approach named cos98 matching [31]. For the cos98 matching, all the tokens in the text receive a weight according to their inverse document frequency across the Medline corpus. The similarity of two annotations is calculated as the cosine similarity between all tokens and their weights in the two annotated strings. A high cos98 (cosine similarity > 0.98) score is achieved if the two annotated strings differ only by terms with a low information content, such as “the” or “protein”. In the case of BioCreative-II, only the gene list has been considered during exact evaluation, and the whole gene list in combination with the alternative gene list has been used for the correlation in the cos98 matching evaluation. All results and descriptions of the used systems are also available through the CALBC League Table [67].

Since the lexical resources – in essence – have been derived from the biomedical reference databases, they are considered to represent the domain knowledge. If a *LexTag* solution produces a low FN error rate on a GSC then we call the tagger *complete* with regards to the corpus. If a *LexTag* solution produces a low FP error rate on a GSC then we call the corpus *complete* with regards to the tagger. If both conditions are true, then we call the tagger *compliant* with the corpus. We call two PGN tagging solutions compliant, if they are compliant against the same corpus. If they only have very similar FP and FN profiles against a corpus, we call them *replaceable*.

### Categorisation of FP and FN results

For better assessment of the FP and FN results, a selection of the 15 most frequent FP and FN results, respectively, for all pairs composed of a corpus and a tagging solution has been made. This approach is suitable for the two following reasons. First, it allows comparing the corpora, although the corpora have different sizes and different annotation standards, and thus they produce different amounts of errors which limit their comparability if all FP and all FN results have been considered. And second, the removal of the most frequent mistakes is a common approach to improve the quality of a tagging solution against a corpus.

We can expect – from a theoretical point of view – that the tagging solutions will produce similar FP results across the different corpora under the assumption that the corpora have been extracted from the same collection of documents. In our case, all corpora stem from Medline abstracts, but the topic of each one of them may differ. On the other hand, we would expect very similar FN results only for any single corpus but not necessarily across all corpora, since the corpora do not exactly follow the same annotation standards.

The terms forming the most frequent FP and FN errors have been manually analyzed and categorized into different sets (see Table 4). The selected categories consider morphological and semantic differences and again induce an abstraction of the findings to support comparability between the different corpus-tagger experiments. Furthermore, the different error types have been sorted according to the categories “Core”, “Margin” and “Artefact” of decreasing relevance for the PGN tagging task. Artefacts are general English terms, expanded a-PG, since they convey a modified semantics, and single characters, which do not sufficiently abbreviate a PGN. Terms in the category BMT, ea-PG and 2C do represent PGNs as generalizations or family terms, as modified a-PGs or intermediary forms between PGT and a-PG, and as very short a-PGs, respectively. The core terms certainly should be fully annotated in a corpus, and should be completely identified by a PGN tagging solution. All FP and FN results have been categorized and the counts have been normalized based on the total number of annotations in a corpus to yield representative and comparable figures.

**Table 4:** This table gives a schema for categorizing terms into different types that help to sort and count the most frequent false positive and false negative results

**Label**	**Description**	**Semantics**	**Relevance**	**Examples**
1C	1 character	Undefined	Artefact	N
2C	2 characters	Undefined	Margin	T3, PU, LH
a-PG	Acronym PGN	Confirmed by UniProtKb	Core	Ras, c-fos, Wnt
ea-PG	Extended a-PG	a-PG with preserved semantics	Margin	Ras gene, p53 protein, Src family
xa-PG	Expanded a-PG	a-PG with modified semantics	Artefact	p53 mutations
PGT	Protein/gene term	Confirmed by UniProtKb	Core	E-cadherin, beta-catenin, glucocorticoid receptor
BMT	Biomedical term	Specific to biomedical scientific text, excluding PGT	Margin	olymerase, prion, IgM
GE	General English term	Occurring in non-scientific text	Artefact	Plasma, renal, inhibitor, antibodies

## Results

In this manuscript, we present the results from a study comparing different PGN tagging solutions against all available GSCs and measuring performances. This research enables a better understanding, what contributions for the tagging performances can be expected from the components of a PGN tagging solution. Furthermore – by analysing the FP and FN errors – we explore the characteristics of the erroneous annotations by the different solutions.

### Comparison of the lexical resources

The lexical resources differ in size and content, and also differ in the terminology that is shared between them when performing comparisons (cf. Table 3). The largest lexical resource, i.e. GP7, comprises 1,725,500 unique terms, whereas the smallest one, i.e. SwissProt, comprises only 228,893. Even the change from one version to the next, i.e. from GP6 to GP7, can lead to a strong increase of unique terms and term variants. Manual inspection has shown that GP7 incorporates a larger number of general English terms as term variants [12]. The different lexical resources are homogeneous in the sense that they share significant portions of their content, and it can be expected that the larger resources improve the recall of *LexTag* solutions.

### Evaluation of taggers across corpora: F1-measures

**Figure 2:**
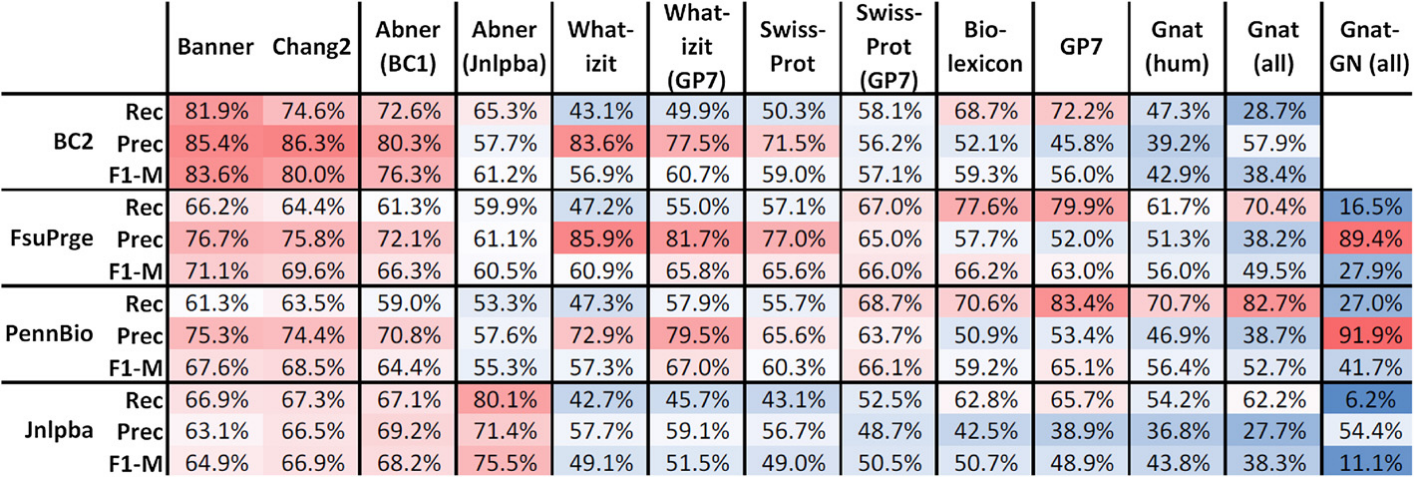
**All taggers have been tested against all GSCs using cos98 similarity for evaluation (see also Figures **4**, **5** and **6**).**

**Figure 3:**
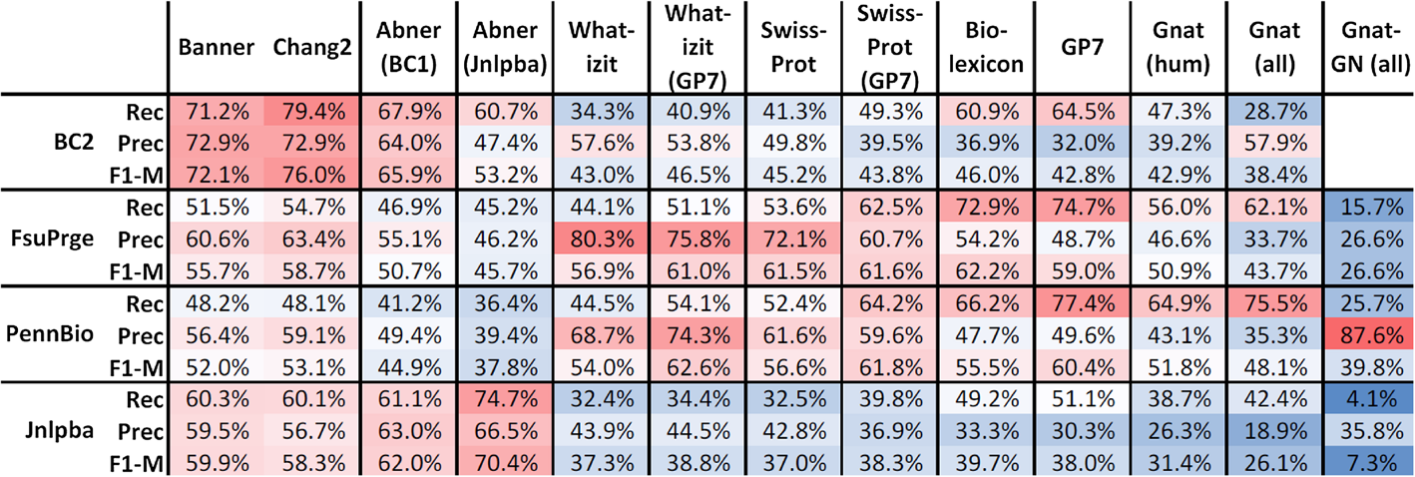
**Again all taggers have been tested against all corpora, but now exact matching has been applied for evaluation.** The table shows the same performance results as Figure 2 with the difference that exact matching has been used as evaluation form (see also Figures 4, 5 and 6).

All supervised solutions show their best performance against those corpora that have been used for their training and lower performances against the other corpora. Banner shows the best performance in comparison to all the other PGN taggers, if we consider BioCreative-II only (exact and cos98 matching evaluation), i.e. Banner is compliant with BioCreative-II (see Materials and methods section). The Chang2 PGN tagger shows similar but slightly lower performance on the same corpus. It can be as well considered compliant with BioCreative-II, but would also profit from optimisation to reach the performance of Banner at the expense of lower precision (cf. Figures 2 and 3).

**Figure 4:**
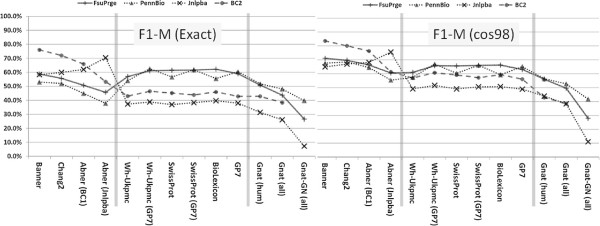
**The diagram shows the F1-measure performance of the different PGN taggers against the selected corpora using exact as well as cos98 evaluation.** The *ML-Tag* solutions have been trained on BioCreative-II (Banner, Chang2), on BioCreative-I (Abner (BC1)) and on Jnlpba (Abner (Jnlpba)) and therefore perform best on these corpora. The *LexTag* solutions show similar performance across all corpora. In the left diagram all solutions have been measured using exact matching against the entity boundaries in the GSCs; for BioCreative-II only the gene list has been used for the evaluation. The measurements in the right diagram use a relaxed measure based on cosine similarity (cos98) between the tagged results and the GSC annotations leading to higher F1-measures; for BC2 the gene list and the alternative gene list has been applied. In both diagrams, the the entries in the left third represent the *ML-Tag* solutions, the middle part the *LexTag* solutions and the right part the Gnat solutions as a reference to a gene normalisation tagger. The performances of the *LexTag* solutions against the corpora reach higher F1-measures for FsuPrge and PennBio than for BioCreative-II and Jnlpba.

On the FsuPrge corpus, Banner performs best but only when cos98 matching is applied, and its performance is still lower than four of the six *Lex-Tag* solutions (for exact matching, cf. Figure 4). On PennBio and Jnlpba, Banner is not the best performing solution and even Chang2 performs better (except for PennBio, exact matching). Note that Abner(BC1) has been trained on BioCreative-I and shows the best performance against the BioCreative-II test corpus indicating that similar annotation guidelines apply to both corpora.

When using cos98 matching – in contrast to exact matching – the performance of the tagging solutions increases (5% to 10%, cf. Figure 4). As a result the performance differences between the individual PGN taggers measured against a single corpus become smaller for all corpora. More in detail, on FsuPrge and PennBio the differences are reduced to less than 10% (cos98 matching), except for the Gnat solutions and for Abner on PennBio.

**Figure 5:**
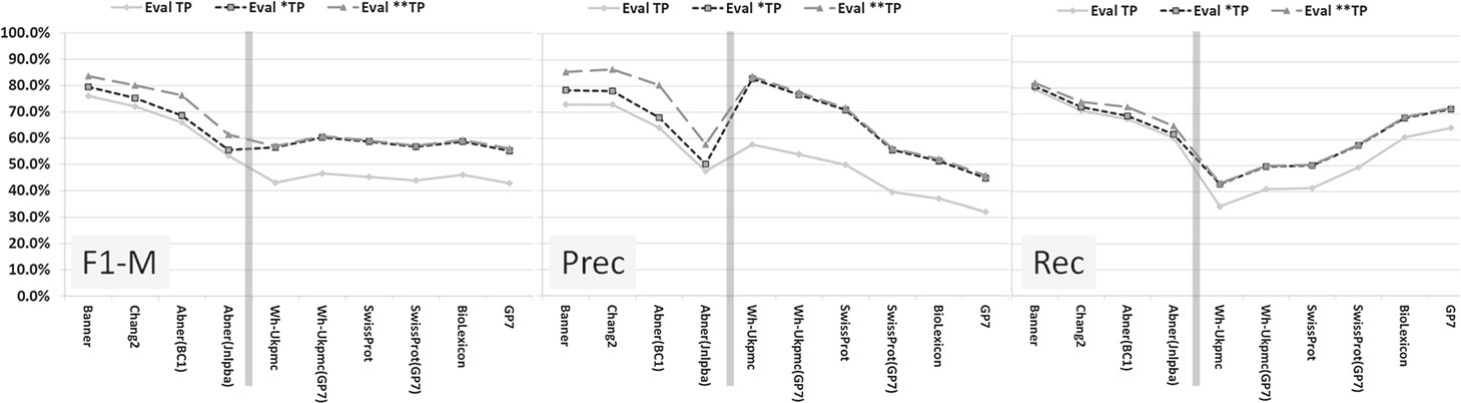
**The three diagrams show the evaluation of the different tagging solutions against the BioCreative-II corpus only.** For the evaluation represented by the solid line only the gene list of the BioCreative-II evaluation kit has been used. For the two other lines also the alternative gene list has been applied counting the '*TP’ (dotted line) and the '**TP’ (dashed line) as TP candidates (instead of FP candidates). From the first to the last evaluation all three measures improve, and only the *ML-Tag* solutions profit from the '**TP’ data entries in the alternative gene list of BioCreative-II. Obviously these candidates are not part of any lexical resource and only occur in the BioCreative-II corpus.

One reason for this change could be that PennBio and Jnlpba follow different naming conventions in comparison to current lexical resources. Cos98 similarity relaxes the importance of term variability in the evaluation against the PGN mention boundaries in the corpus. This has already been acknowledged in the BioCreative-II challenge through the introduction of an alternative gene list, which accomplishes the regular gene list (cf. Figure 5) [23].

When considering the alternative gene list, the performance of the tagging solutions increases similar to the cos98 evaluation on the other corpora. Selected data entries on the alternative gene list, which are marked as '**TP’ in the evaluation result, are only recognized by the *ML-Tag* solutions'and may have been added at a different time point than the other entries.

The performance of GNAT is lower than the one from the other solutions. This result is expected, since GNAT performs gene normalisation, i.e. it anticipates assigning a small selection of identifiers or a single identifier to the protein mention, which is a more complex task than gene mention annotation and automatically leads to reduction in recall.

### Precision and recall evaluations

**Figure 6:**
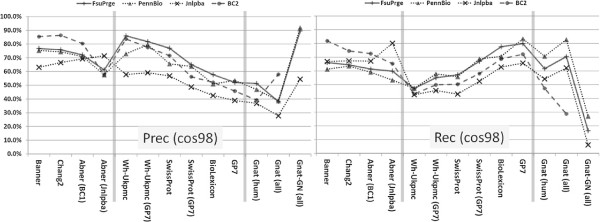
**The precision and recall performances in these diagrams are based on cos98 evaluation.** The left and the right diagram give an overview on the precision and recall measurements of the PGN taggers against the selected GSCs. For *ML-Tag* methods both parameters are best, if the taggers have been tested against those corpora that they have been trained on. The *LexTag* methods have been sorted from left to right according to their precision performance with the best performing solution to the left. With minor deviations, all *LexTag* methods show best performances against the FsuPrge corpus for both, precision and recall. The “trade-off” between precision and recall for methods with similar F1-measures is well known, but it is still remarkable that the *LexTag* solutions can differ considerably on their precision and recall values, whereas the F1-measure seems to be almost invariable across them. Again, precision and recall performances of the *LexTag* solutions against the corpora reach higher values for FsuPrge and PennBio than for BioCreative-II and Jnlpba.

When comparing the precision and recall performance of the tagger (cf. Figure 6), both Banner and Chang2 show the best performance against the BioCreative-II test corpus. The best performing *LexTag* solutions either show high recall, in particular if they incorporate the latest version of the Biothesaurus (GP7), or high precision if they use either a small lexical resource, for example the SwissProt tagger, or if they make use of efficient FP filtering as it is the case for Wh-Ukpmc. Again, cos98 matching gives higher precision values than exact matching indicating variability in the gene mention boundaries (see BioCreative-II, alternative gene list).

Comparing the PGN taggers across the different corpora leads to the conclusion that the performance on one corpus is predictive for the others (with a few exceptions), i.e. the order of corpora with regards to the performance is preserved across the different taggers, keeping in mind that the *ML-Tag* solutions perform best on the test corpora of the GSC used in their training. It is also remarkable that the recall performances of FsuPrge and PennBio are less than 5% apart across the different tagging solutions (apart from Abner (Jnlpba) and BioLexicon), indicating that PennBio and FsuPrge may comply better with the naming guidelines for gene entities.

### Comparing FP and FN profiles between tagging solutions

#### ***FN results are indicative for the corpus***

**Figure 7:**
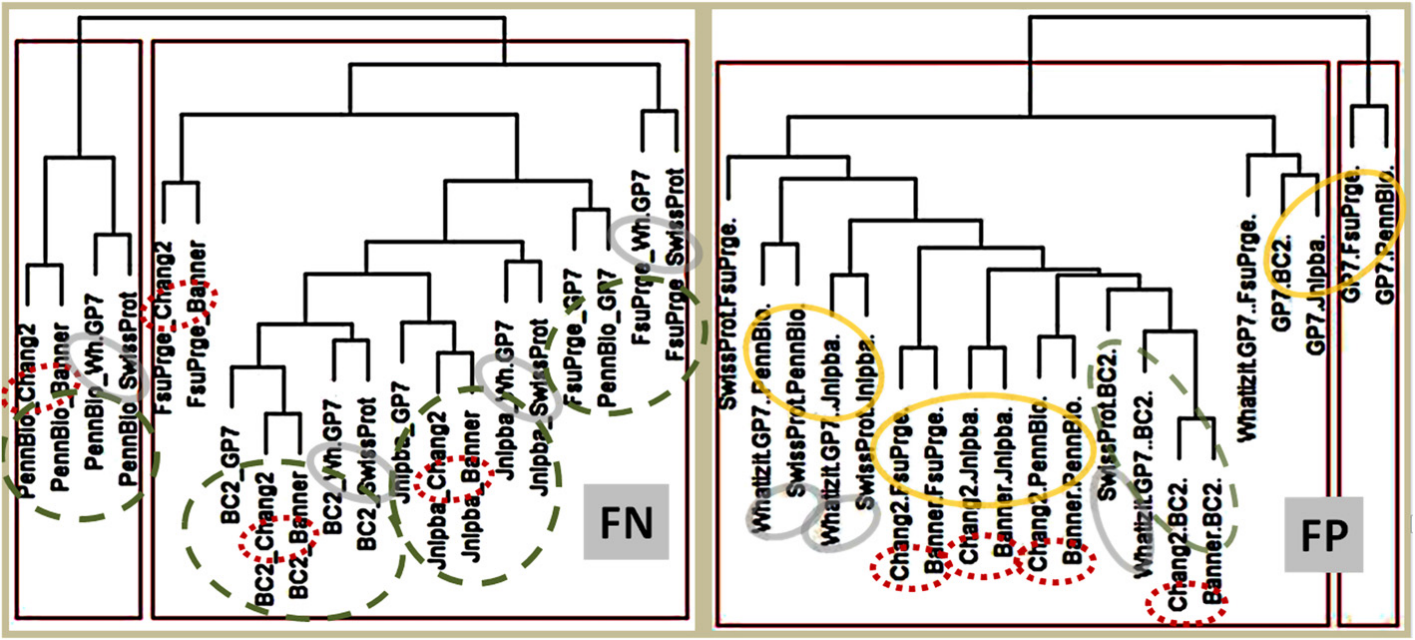
**Clustering has been applied to determine the similarity of the tagging solutions based on the FP and FN profiles determined on the different GSCs.** The most frequent 100 FNs and FPs, respectively, from all annotations for a given corpus have been used to characterise the performance of a PGN tagger against the different corpora (GP7: GeneProt7, Wh.GP7: Wh-Ukpmc (GP7)). Four taggers have been selected that are closely related due to their components. The comparison of their FP and FN errors, respectively, or their FP and FN profiles, will help to trace back types of errors to the composition of the tagging solutions. The error profiles have been clustered according to their FN profile similarity (left diagram) and their FP profile similarity (right diagram). – Chang2 and Banner profiles always cluster together, since they rely on the same technology. The FN profiles of the taggers cluster together according to the corpus which has been used to test them, since the FN errors are predefined by the annotation standards of the corpus, i.e. by the pre-assigned annotations missed by a tagger. When we take the FP profiles into consideration, the taggers cluster together across the different corpora. Here, it is also remarkable that SP and Wh7 cluster together, although they do differ in terms of their resources.

The error profiles of four taggers have been analysed to explore the response of the taggers against the GSCs. The FN errors of the annotations have been extracted and compared (exact evaluation, cf. Figure 7). The results from the tagging solutions can be compared due to the reuse of similar lexical resources at different sizes and the reuse of the Chang2 tagger as FP filter. As mentioned above the comparison is done based on hierarchical clustering (see Materials and methods section).

Since the FN results are defined by the corpus annotation standards, the taggers cluster together according to the corpus which they have been tested on, i.e. the tagging solutions produce similar FN results and can be called complete towards a given corpus if they produce a small number of FN results (see Materials and methods section). In addition, the FN results of the Chang2 tagger and Banner are very similar, since both have been trained on the same corpus, but the FN errors of SwissProt and Wh-Ukpmc(GP7) cluster as well together. Both have very similar precision and recall performances, and include similar lexical resources.

**Figure 8:**
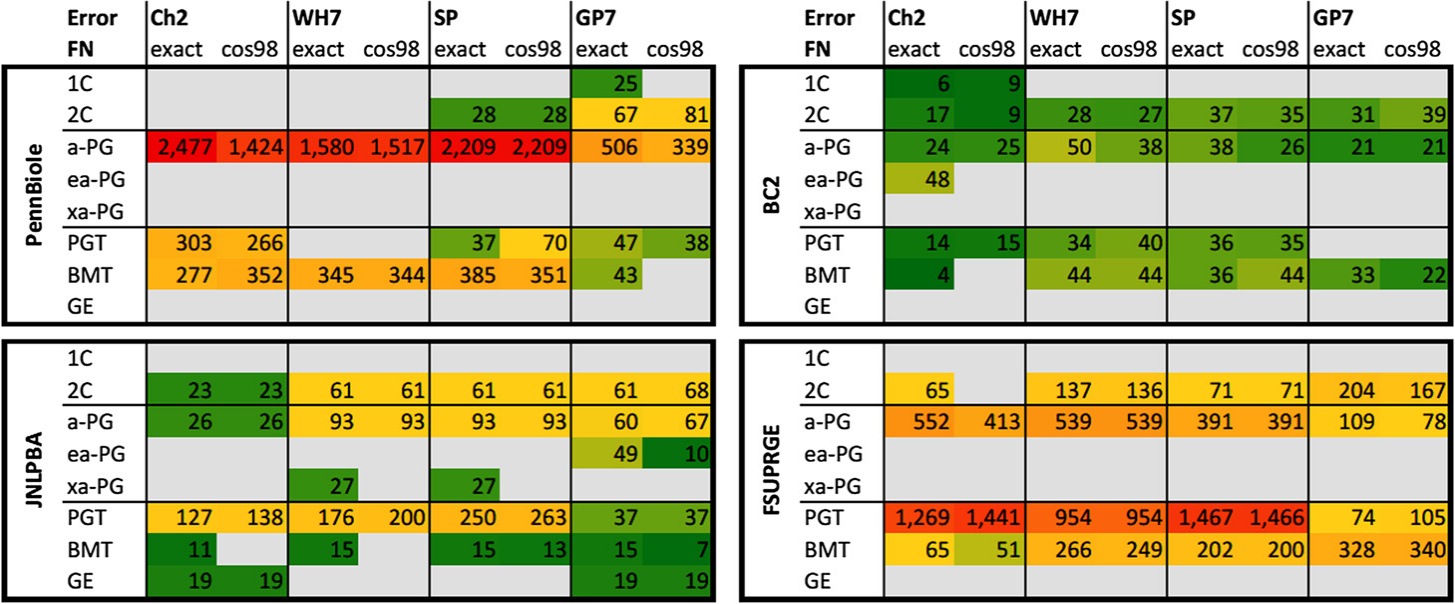
**The diagram gives an overview on the most frequent FN results.** The FN results have been categorized according to the morphology and the semantics of the missed terms. For all GSCs and the different tagging solutions the counts for the 15 most frequent FN errors are shown. Since PennBio and FsuPrge are the largest GSCs, they induce also a larger number of FN results. FN results of GE terms are mainly encountered in Jnlpba. FsuPrge generates a larger portion of PGT FN results, whereas PennBio mainly leads to a-PG FN results.

A more detailed analysis of the FN errors is given in Figure 8: the 15 most frequent FN errors for each corpus-tagger pair have been manually categorized into different groups according to their morphological and semantic criteria (see Materials and methods section). Three kinds of FN errors dominate the outcome: (1) missed acronyms (a-PG), (2) missed long-form PGNs (PGT) and (3) missed unspecific biomedical terms (BMT).

GenProt 7.0 (GP7) has the highest recall and thus the lowest number of FN errors due to its large terminological resource. Considering PennBio, the other three taggers miss mainly the acronym forms of the PGNs, which hints towards completeness of the corpus with standard acronyms. For other corpora, i.e. for FsuPrge and Jnlpba, the FN errors fall into the PGT category, which in general show high diversity.

Furthermore, the error profiles for FsuPrge across all tagging solutions show high similarity indicating that this corpus is complete in its annotation and thus robust against alternative tagging solutions. PGN tagging solutions have the lowest number of FN errors on the BioCreative-II corpus, indicating that this corpus is complete with regards to the tagging solutions. On the other hand, the SwissProt tagger generates across all corpora the same FN errors and is thus more consistent across all the corpora.

**Figure 9:**
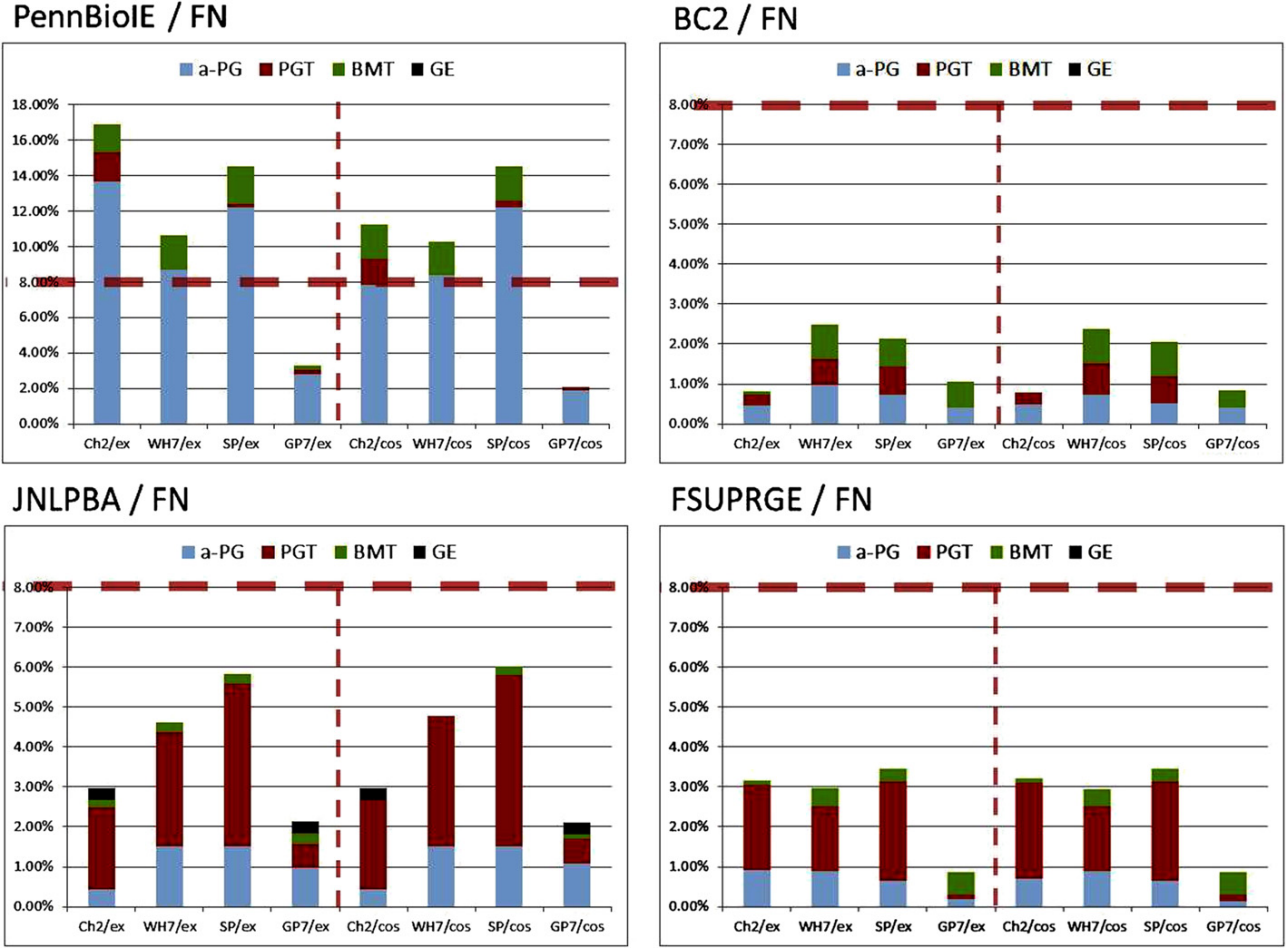
**The diagram displays the portions of FN results of the tagging solutions against the GSCs.** The figure visualises the results from Figure 8, where the error frequencies have been normalized by the annotations contained in the GSC. The FN errors in Jnlpba and in FsuPrge are frequently of type PGT, whereas the other two corpora rather produce a-PG FN results. PennBio and Jnlpba appear to be less compliant with the terminological standards of the tagging solutions than the other two corpora. Exact matching and cos98 matching produce very similar results, excluding that the selection of annotation boundaries could be the main source of the FN results.

The four diagrams in Figure 9 give an overview on the normalized distribution of FN errors across the four different corpora. Jnlpba is the only corpus generating general English (GE) FN mistakes indicating that Jnlpba deviates from the standard in the other corpora, but all corpora do induce FN results that are categorized as biomedical terms (BMT). This category gathers those terms that are considered to represent less specific terms than PGN mentions. Cos98 matching partially resolves boundary mismatches, and therefore reduces the FN error rates in PennBio, in particular for Chang2 and GP7, but also in BioCreative-II.

Overall, it can be concluded that BioCreative-II and FsuPrge are better compliant with the naming conventions in the biomedical domain than the other two corpora, i.e. induce FN errors due to GE and BMT terms at a lower rate. In particular FsuPrge generates comparable FN profiles across the different tagging solutions, indicating that less common PGT and a-PG terms are difficult to capture for all tagging solutions.

On the other side – as pointed out before – all corpora are predictive for the performance of the tagging solutions alike (see above).

#### ***FP results are indicative for the tagging solutions***

FP errors represent those PGN mentions that have been identified by the tagging solution but do not match the annotations in the GSCs. Collecting and analyzing the set of false positives for each tagger and corpora pairs gives further insight for the types of errors produced by each tagger and for potential deficits in the annotation standards for the corpora. Clustering the FP profiles of the tagging solutions (cos98 matching, cf. Figure 7) reveals that – as expected – Chang2 and Banner produce similar FP results. Apart from this, Wh-Ukpmc(GP7) and SwissProt cluster together on the different corpora because they use similar terminological resources. GenProt 7.0 is clearly distinct from the other tagging solutions due to its high false positive rate.

The Pearson product-moment correlation coefficient has been determined for the different pairs, where each pair is composed of a PGN tagger and a corpus. The correlation calculations are based on the false positives results produced by the combination of a PGN tagger and a corpus (see Figure S9, in the Additional file 1). The correlation matrix shows the pair-wise similarity of the taggers across the different corpora with the strongest correlations given between Wh-Ukpmc(GP7) and SwissProt on PennBio, BioCreative-II and Jnlpba, and between Chang2 and Wh-Ukpmc(GP7) across the different corpora. This could be due to the use of the same FP filtering in both solutions. Again, GeneProt7.0 (GP7) is clearly distinct due to the lack of efficient FP filtering.

**Figure 10:**
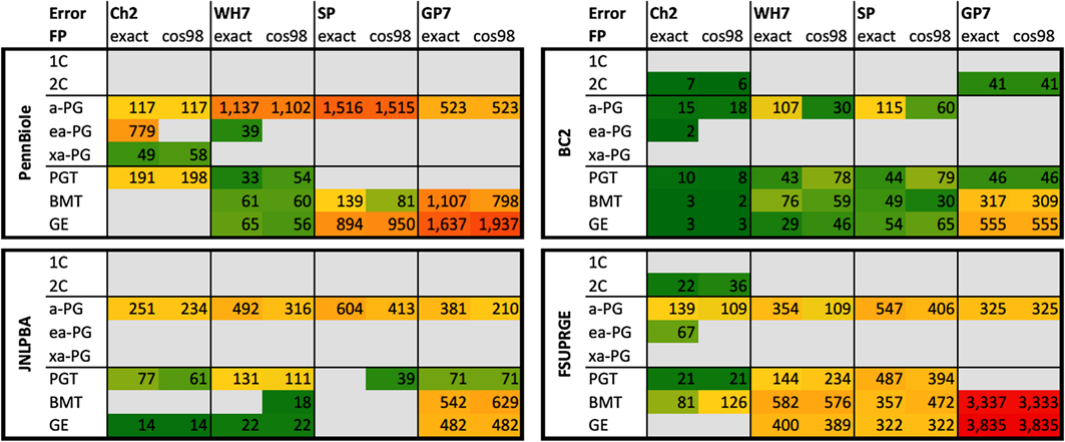
**The overview lists the most frequent FP results according to predefined categories.** The FP results have again been categorized according to the morphology and the semantics of the missed terms (see Figure 8 above). Again, for all GSCs and the different tagging solutions the counts for the 15 most frequent FP errors are displayed. GP7 and Wh-Ukpmc (GP7) are based on a very large terminological resource that generates BMT and GE FP errors in larger numbers. FP filtering with Chang2 reduces the rates in the case of GP7. The profile of Chang2 differs from the others in the sense that it generates ea-PG and xa-PG FP errors increasing acronyms to larger term structures.

The Chang2 tagger generates the smallest number of false positives, thus showing high precision across the different corpora. Its FP results can be grouped into acronyms (a-PG) or long-form (PGT) PGNs. On the other hand, the GP7 tagger generates a significant amount of false positives that can be classified as other biomedical terms and general English terms (see Figure 10). This is in particular true for the FsuPrge corpus indicating that most a-PG and PGT terms have been identified.

SwissProt and Wh-Ukpmc (GP7) show very similar results in FsuPrge and BioCreative-II again indicating that both corpora follow the same naming standards; and both show a slightly more complementary distribution of FP errors in Jnlpba and PennBio. This result shows that Wh7 produces a higher number of FP errors in the categories BMT and GE, which cannot be compensated through false positive filtering, since Chang2 is optimized for long-form and acronym PGNs in contrast to other biomedical terms.

From a methodological point of view, Wh-Ukpmc(GP7) has the advantage of a large lexical resource in combination with a precise *ML-Tag* solution for FP filtering, however, SP performs evenly well, although the terminological resource in SP has been kept to the standard of previous years. The best explanation is that the lexical resource of SwissProt incorporates the “conserved” part of the different terminological resources and generates only a small number of FP results.

**Figure 11:**
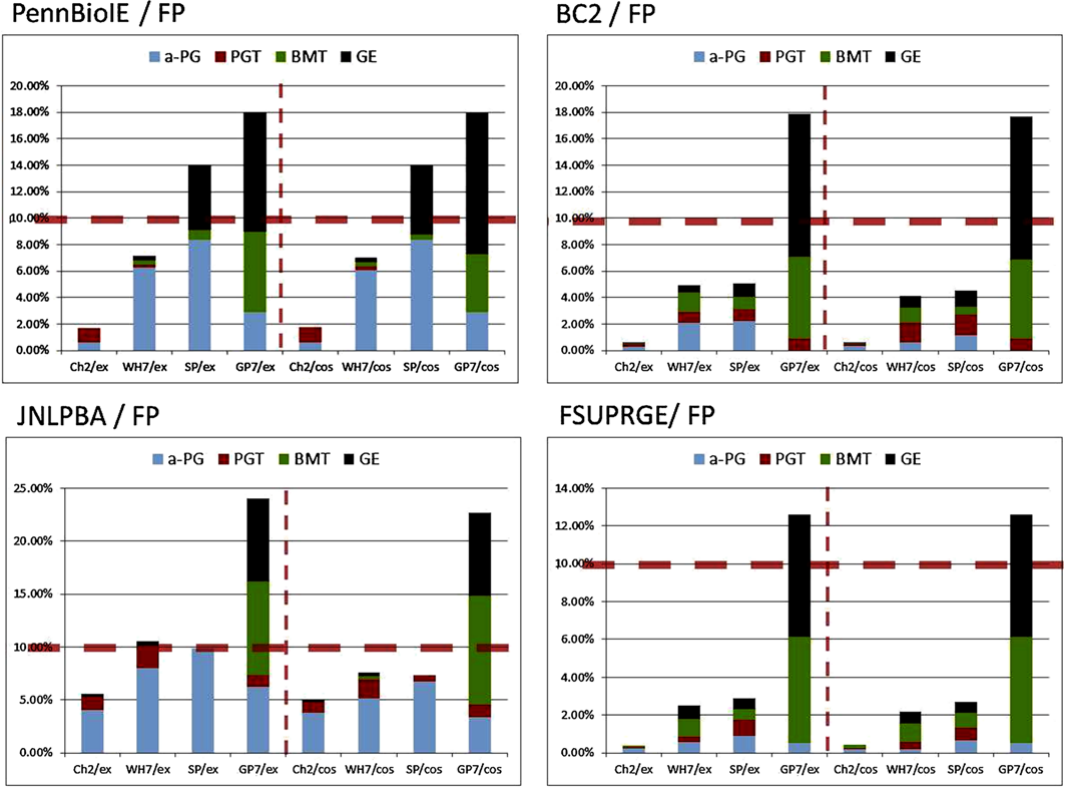
**The diagram displays the FP results of the tagging solutions in form of error frequencies normalized against the annotation numbers in the GSC.** As expected, GP7 produces lot of FP errors in all corpora which have been reduced in Wh-Ukpmc (GP7) through FP filtering using Chang2. All solutions produce larger numbers of FP errors in Jnlpba and PennBio leading to the conclusion that both corpora are less compliant with the current terminological standards in the tagging of PGNs.

Figure 11 shows the normalized FP errors taking the number of annotations into consideration. Taggers on the FsuPrge corpora produce the smallest FP error rates with exception of the GP7 tagging solution (above 12%), and BioCreative-II generates a similar result with a lower error rate for GP7 (below 10%). This difference can be explained by the fact that FsuPrge covers full abstracts whereas the BioCreative-II corpus is composed of single sentences, which increases the rate of specific terms over unspecific terms in the corpus. PennBio and Jnlpba include a higher FP error rate for a-PG and even the Chang2 tagging solution has a FP error rate of above 5% on the 15 most frequent FP errors showing that Jnlpba follows slightly different naming conventions than the other corpora.

#### ***FP and FN error distribution according to their relevance***

The last analysis qualifies the FP and FN mistakes into categories, where each category is attributed a level of relevance for the PGN tagging task. The idea behind this approach is the observation that it is of higher importance to identify specific types of PGNs, e.g. the acronyms and the long-form mentions of PGNs (a-PG and PGT), in contrast to other mentions of PGNs, e.g. single character PGNs and terms representing general English concepts. In a more strict sense, if a corpus represents well the best conserved PGN terms and a tagging solution identifies them at a high performance measure, then this solution would fulfill the tagging task more reliably in comparison to either a corpus that has a higher variety of PGN annotations and misses out on well conserved PGN terms, or in comparison to a PGN tagging solution that rather identifies less specific terms (e.g., GE or BMT terms) or modifications of a-PG terms (e.g. ea-PG or xa-PG) in contrast to the most conserved PGN terms.

**Figure 12:**
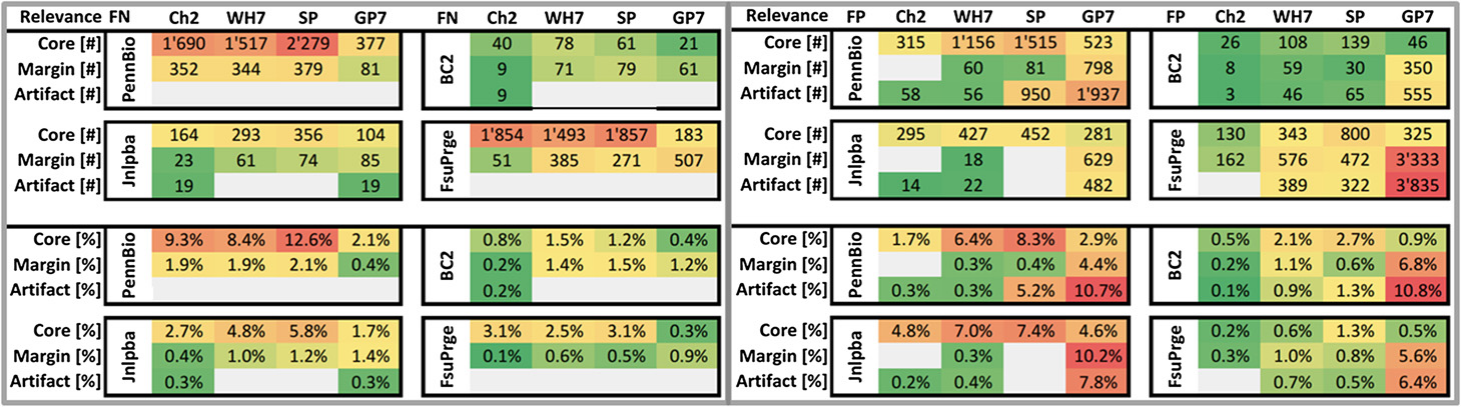
**The error in the tagger-corpora pairs have been categorized according to their relevance.** The error types and counts from Figures 8 and 10 have been grouped into the categories “Core” (for a-PG and PGT), “Margin” (for 2C, ea-PG, BMT) and “Artefact” (for 1C, xa-PG, GE) representing different levels of relevance for the PGN tagging task. The FN results are on the left, the FP results are to the right, the upper part contains the absolute numbers and the lower figures have been normalized according to the total number of annotations in the corpora. Only the results for the cos98 evaluation are shown.

When regarding the FN results (cf. Figure 12), it becomes clear that only very few FN errors can be found that are classified as artifacts. We can expect that the curators have excluded annotations that would be categorized as artifacts, since this type of annotations are unspecific for PGNs. On the other side, the tagging solutions do produce FP errors in this category including the *ML-Tag* solution Chang2, and most FP errors occur if a large terminological resource is applied unfiltered (see GP7 in Figure 12).

When analysing the annotations that are classified as “Core”, we discover that the biggest number of FN results occur in absolute and relative numbers on PennBio even for different tagging solutions, indicating that the annotations may not be conform with the standard biomedical data resources (non-compliant annotations). When considering Wh-Ukpmc (GP7) and SwissProt, a high portion of FP annotations belonging to the category “Core” can again be found for PennBio and Jnlpba, which indicates that not all PGN mentions have been annotated.

For the GP7 annotation solution we find that it over-generates annotations due to the lack of FP filtering for all corpora. On the other side, the BC2 corpus and FsuPrge do annotate the domain terminology well (inducing a small number of FN results), and also capture all PGN findings in the corpora, leaving little room for FP mistakes.

## Discussion

The presented analysis gives an overview on the different PGN tagging solutions against the GSCs. As expected, we can judge the tagging solutions against the GSCs, but – in principle – we can also judge the GSCs against the tagging solutions. For the former case, we learn that the annotations in the GSCs differ from each other leading to different FN profiles when measuring the performance of the taggers against the corpora. From the FP profiles, it is possible to derive that the corpora differ in their degree of completeness with regards to the tagging solutions.

### 

#### ***Judging the tagging solutions***

According to the FP rate of the taggers against the different GSCs allows for a better understanding of their working and propose approaches for their improvement. For example, the GP7 *LexTag* solution annotates a larger number of general English terms, e.g. “kinases”, which may or may not be relevant for the overall annotation of gene entities, but which in general is not included in the annotations of the different corpora (cf. Additional file 1: Tables S12 and S13).

As expected, the *ML-Tag* solutions perform better on the test set of the GSCs used for their training data, however they do show mediocre performance on the other corpora. In addition, the *ML-Tag* approaches do not deliver any clues for the normalization of the identified protein or gene entity. The biggest advantage of the *ML-Tag* approaches lies in the context-sensitive disambiguation of entities. This characteristic has been exploited in our experiments to improve the results of the *LexTag* solutions through FP filtering and showed the beneficial positive results. It became also clear that this FP filtering did not effectively work for general biomedical and general English terms, which were not recognized as FP annotations.

Applying FP filtering to the *LexTag* solutions increases precision and reduces recall as shown by our experiments, however it is not obvious what the best combination should be. More in detail, the different *LexTag* approaches produced very similar F1-measure performances, although they differ in their FP and FN profiles. A priori, it is an advantage to use a large lexical resource to increase the recall, but as can be seen from our results this approach largely reduces the precision. Fortunately, in the case of high recall and low precision, it is possible to apply FP filtering to the *LexTag* solution for lowering high recall, improving low precision and eventually the F1-measure performance after all.

The *ML-Tag* approaches reproduce the corpus annotations and have the following characteristics: (1) they can identify terms that might not be included in any lexical resource, (2) can cope with terminological variability, and (3) might still ignore a gene mention, if the context lacks to deliver the right clues. The combination of *LexTag* and *ML-Tag* solutions give the opportunity to combine efficient gene mention identification with gene normalization, and gene mention methods should perform better than gene normalization, since not all gene mentions are known to the lexical approach.

#### ***Judging the GSCs***

The PennBio corpus and the FsuPrge corpus are the two corpora where the taggers tend to deliver their best performance. This could be explained by the fact that both corpora have been created more recently than the BioCreAtIve-II and the Jnlpba corpus and therefore they may better comply with naming conventions in the biological domain, i.e. comply better with latest annotation guidelines for genes and proteins.

The analysis of the FP and the FN errors has demonstrated that BioCreative-II and FsuPrge are more compliant with the terminological resources than the other two corpora are. It is surprising that the tagging solutions perform worse on the BioCreative-II corpus than on the FsuPrge corpus, but this can be explained by the fact that the single sentences in the BioCreative-II corpus have been selected in a way which is very specific to biomedical text mining, or – the other way around – the context in the FsuPrge full Medline abstracts contains a higher diversity of non-specific terms which reduces the performance of the tagging solutions.

When comparing the evaluation based on exact matching to the one from cos98 matching, the performance of all tagging solutions improves, but their relative performance against the different corpora stays the same, i.e. the order of the corpora measured by the performance against any given tagger is unchanged. In particular, the performance of the tagging solutions measured against Jnlpba and BioCreative-II improve when moving from exact to alias matching. This shows that these two corpora comprise more term variability in their annotations in comparison to the other two corpora, in particular since both corpora are smaller in their size.

The annotated corpora and the used terminological resources have been produced at different time intervals. As a result, a lexical resource from the past should be more compliant with a corpus that is outdated as well. This assumption could not be confirmed. It is rather the case that a larger lexical resource contains more spurious terms, generates more noise induced by the FP errors in the tagging results, and that other parameters are more relevant for the terminological variability in the corpora such as the annotation guidelines. Although the distinction between gene acronyms only and long-form PGNs on the one side, and between the genes in general and biomedical terms or general English terms can be difficult, we could identify that the identification of a-PG terms is best performed with a *ML-Tag* solution whereas the other forms are better identified with *LexTag* solutions including morphological or even syntactical variability.

## Conclusion

The following findings can be drawn from our study. The *ML-Tag* approaches work best on the corpus where they have been trained on, which has been expected in the first place. Chang2 has been trained on BioCreative-II and performs best on BioCreative-II. Abner (BC1) used the BioCreative-I corpus and shows best performance on BioCreative-II. Abner (Jnlpba) has its best results on Jnlpba where it has been trained on. Unfortunately, their performances drop when moving to a different corpus. These results show that the tagger performances highly depend on the corpora that they have been trained on and thus they cannot generalize well from the learning corpora to the other corpora.

The *LexTag* solutions have different profiles for their precision and recall performances, but the F1-measure remains in a very similar range. This result is surprising and suggests that they all cover a portion of the best known naming standards, but cope differently with the term variability across the corpora.

The FP filtering does improve the results by increasing the precision of the *LexTag* solution without compromising the recall too extensively. This improvement is best if the *LexTag* solution delivers high recall already at the expense of low precision. Nonetheless, this approach still leads to lower performance in comparison to *ML-Tag* solutions only, which is again due to the fact that the *ML-Tag* solutions are performing better on the context-sensitive disambiguation and selection of annotation boundaries.

Finally, the topic and the structure of the corpus play an important role. BioCreative-II has a very narrow focus to specific biomedical sentences, which differs from the language in the other three corpora where contextual non-biomedical terms increase the FP error rate. This has to be considered when optimising tagging solutions against the given corpora.

As an overall conclusion, we suggest that the right selection of a *LexTag* solution can help to optimize the text mining output either for higher precision or for higher recall. If the best possible balanced F1-measure is required, then the *ML-Tag* solutions would be the best choice, but it would have to be trained on all available corpora [68]. The combination of a *LexTag* solution and a *ML-Tag* solution for FP filtering is in addition a performing approach.

## Endnotes

^a^http://www.calbc.eu

^b^http://cbioc.eas.asu.edu/banner/

^c^http://www.natcorp.ox.ac.uk/

^d^http://stat.ethz.ch/R-manual/R-patched/library/stats/html/hclust.html

## Competing interests

The authors declare that they have no competing interests.

## Authors’ contributions

AJY, SK, JHK and CL have performed – with different contributions – the experiments to the research work. AJY has developed the core IT components to enable the alignment of corpora. SK has thoroughly tested the taggers against the corpora. JHK, RH and RB have contributed to the evaluation of the results. DRS has performed the statistical analysis and has written the manuscript. All authors read and approved the final manuscript.

## Supplementary Material

Additional file 1 Supplementary Material. Click here for file
