# Supplementary material for: Evaluating gold standard corpora against gene/protein tagging solutions and lexical resources
Source: J Biomed Semantics. 2013 Oct 11;4:28. doi: 10.1186/2041-1480-4-28 (PMC4021975; doi:10.1186/2041-1480-4-28)
Supplement: Additional file 1 — Supplementary Material. [file 2041-1480-4-28-S1.pdf]

Supplementary Material

Correlation matrices on FP and FN profiles

The following two tables give the correlation coefficients for the FP profiles and the FN profiles of a single tagging solutions against a selected GSC (using cos98 evaluation).

- Fig. 8: Correlation matrix for the FN errors.
- Fig. 9: Correlation matrix for the FP errors.

		FsuPrge			BC2				PennBio				Jnlpba			
		Wh7	SwissProt	GP7	Chang2	WH7	SwissProt	GP7	Chang2	WH7	SwissProt	GP7	Chang2	WH7	SwissProt	GP7
FsuPrge	Chang2	0.35	0.20	0.02	-0.07	-0.15	-0.14	-0.11	-0.01	-0.12	-0.12	-0.13	0.09	-0.03	-0.05	-0.15
	Wh7		0.40	0.12	-0.17	0.03	-0.01	-0.20	-0.08	-0.15	-0.11	-0.15	-0.03	0.03	-0.13	-0.20
	SwissProt			0.04	-0.17	-0.02	0.18	-0.13	-0.13	-0.09	-0.10	-0.13	-0.14	0.00	-0.02	-0.14
	GP7				-0.11	0.03	-0.05	0.05	-0.13	-0.13	-0.13	0.05	-0.09	-0.15	0.03	-0.07
BC2	Chang2					0.10	0.06	0.28	-0.08	-0.16	-0.15	-0.11	-0.13	-0.16	-0.15	-0.12
	WH7						0.34	0.21	-0.14	-0.18	-0.11	-0.04	-0.15	0.04	-0.07	-0.15
	SwissProt							0.10	-0.17	-0.18	-0.08	-0.12	-0.18	-0.05	-0.11	-0.18
	GP7								-0.11	-0.16	-0.11	-0.07	-0.16	-0.20	0.00	-0.08
PennBio	Chang2									0.52	0.34	0.34	0.01	-0.09	-0.09	-0.14
	WH7										0.63	0.39	-0.19	-0.24	-0.19	-0.19
	SwissProt											0.33	-0.09	-0.23	-0.07	-0.17
	GP7												-0.14	-0.15	-0.09	-0.13
Jnlpba	Chang2													0.31	0.24	0.35
	WH7														0.33	0.21
	SwissProt															0.29

Figure 8: **(Correlation matrix for FN profiles):** The matrix shows the pair-wise correlation of the FN error profiles from the different tagging experiments. Each listed PGN tagging solution has been tested on the available gold standard corpora and the FN results from the experiment each tagger-corpus pair have been collected, normalized and then compared. The highest correlation values are given for different PGN taggers against the same corpora.

		Chang2			WH7				SwissProt				GP7			
		BC2	PennBio	Jnlpba	FsuPrge	BC2	PennBio	Jnlpba	FsuPrge	BC2	PennBio	Jnlpba	FsuPrge	BC2	PennBio	Jnlpba
Chang2	FsuPrge	-0.03	0.08	0.07	0.16	-0.03	0.03	0.02	-0.17	-0.17	-0.16	-0.16	0.12	-0.12	-0.03	0.01
	BC2		0.00	-0.08	0.02	-0.06	0.11	-0.05	-0.11	-0.11	-0.02	-0.11	0.05	0.22	0.13	-0.10
	PennBio			-0.11	-0.06	-0.10	0.21	-0.12	-0.16	-0.16	-0.02	-0.15	-0.11	-0.10	0.19	-0.04
	Jnlpba				-0.06	-0.12	-0.16	0.36	-0.18	-0.18	-0.17	0.08	-0.09	-0.09	-0.14	0.19
Wh7	FsuPrge					0.32	0.23	0.15	-0.06	-0.07	-0.11	0.05	0.01	-0.10	-0.08	-0.08
	BC2						0.20	-0.01	-0.06	0.02	-0.16	-0.01	-0.14	-0.04	-0.10	-0.08
	PennBio							-0.02	-0.08	-0.11	-0.07	-0.05	-0.03	0.12	0.37	-0.12
	Jnlpba								-0.19	-0.16	-0.22	0.08	-0.07	-0.11	-0.11	0.39
SwissProt	FsuPrge									0.38	0.31	0.07	-0.20	-0.17	-0.15	-0.19
	BC2										0.28	0.06	-0.07	-0.10	-0.10	-0.23
	PennBio											0.10	-0.01	-0.06	0.16	-0.18
	Jnlpba												-0.15	-0.18	-0.10	0.06
GP7	FsuPrge													0.49	0.31	0.17
	BC2														0.21	0.03
	PennBio															0.02

Figure 9: **(Correlation matrix for FP profiles):** The matrix has been generated in the same way as the correlation matrix in fig. 8, but it now contains the FP error profiles. The table is sorted according to the tagging solutions and the correlation between two tagger-corpus pairs tends to be positive, if the same tagger has been applied to two different corpora.

Tables for FP and FN errors

The following tables list the most frequent FP and FN errors for selected tagging solutions.

- Fig. 10: FN results using exact matching.
- Fig. 11: FP results using exact matching.
- Fig. 12: FN results using cos98 matching.
- Fig. 13: FP results using cos98 matching.

<table>
<tr><th></th><th></th><th>FN - FsuPrge</th><th>FN - PennBio</th><th>FN - BC2</th><th>FN - Jnlpba</th></tr>
<tr><td>exact</td><td>Chang2</td><td>312|KIR, 278|cytokine, 215|cytokines, 203|kinase, 176|transcription factor, 165|transcription factors, 105|RNA polymerase, 74|protease, 71|APP, 65|HDL</td><td>586|K-ras, 385|ras, 329|p53, 303|beta-catenin, 277|polymerase, 252|N-ras, 200|MYCN, 187|N-myc, 115|c-kit, 100|APC</td><td>8|neu, 5|GTPase-activating protein, 5|CREM, 5|LH, 5|chloramphenicol acetyl-transferase, 4|CBF, 4|Ras, 4|E2, 4|CH, 4|Lp</td><td>39|cytokines, 27|transcription factors, 16|glucocorticoid receptor, 15|GR, 14|cytokine, 13|glucocorticoid receptors, 11|antibodies, 11|LTR, 11|proteasome, 10|Glucocorticoid receptors</td></tr>
<tr><td></td><td>Wh7</td><td>278|cytokine, 233|KIR, 215|cytokines, 141|kinase, 131|transcription factors, 119|transcription factor, 108|Tat, 105|IL-1, 103|IL-12, 98|CD8</td><td>450|N-myc, 404|N-ras, 273|polymerase, 157|c-kit, 136|MYCN, 81|VIP, 77|K-ras, 55|KIT, 53|N-RAS, 51|p73</td><td>20|CAT, 15|IgG, 12|c-Jun, 11|IgM, 10|IgA, 10|PU, 9|GH, 9|LH, 9|PI 3-kinase, 9|neu</td><td>69|NF-kappa B, 46|GR, 39|cytokines, 35|Tax, 27|IL-2 promoter, 26|transcription factors, 17|IL-12, 15|nuclear factor-kappaB, 15|ER, 15|AP-1 site</td></tr>
<tr><td></td><td>SP</td><td>278|cytokine, 215|cytokines, 203|kinase, 188|NF-kappaB, 176|transcription factor, 172|RNA polymerase, 165|transcription factors, 108|Tat, 105|IL-1, 103|IL-12</td><td>902|K-ras, 450|N-myc, 404|N-ras, 277|polymerase, 181|H-ras, 81|VIP, 63|Wnt, 53|N-RAS, 46|c-fos, 38|kinase</td><td>15|IgG, 12|c-Jun, 11|IgM, 10|IgA, 10|PU, 9|E2, 9|GH, 9|LH, 9|RXR, 9|PI 3-kinase</td><td>86|NF-kappaB, 69|NF-kappa B, 46|GR, 39|cytokines, 35|Tax, 27|IL-2 promoter, 27|transcription factors, 17|IL-12, 15|nuclear factor-kappaB, 15|ER</td></tr>
<tr><td></td><td>GP7</td><td>108|Tat, 71|Ig, 66|AR, 64|LDL, 47|sigmaK, 44|transcription factor, 40|sigma B, 40|SF-1, 38|gag, 36|sigma K</td><td>124|K-ras, 98|ras, 81|VIP, 69|p53, 52|N-myc, 47|beta-catenin, 41|N-ras, 28|T3, 25|N, 24|telomerase</td><td>9|GH, 9|LH, 8|SH3, 7|SH2 domains, 6|Tat, 6|gag, 6|TCR, 5|NF-Y, 5|Rb, 4|Tax</td><td>46|GR, 35|Tax, 27|IL-2 promoter, 15|ER, 15|AP-1 site, 14|TCR, 12|IL-2 gene, 12|Gadd45gamma, 11|antibodies, 11|LTR</td></tr>
</table>

Table 10: **FN tagging errors (exact)** The table gives an overview on the most frequent errors that occurred when applying the tagger against the corpus. The evaluation is based on exact matching.

<table>
<tr><th></th><th></th><th>FP - FsuPrge</th><th>FP - PennBio</th><th>FP - BC2</th><th>FP - Jnlpba</th></tr>
<tr><td>exact</td><td>Chang2</td><td>35|p38, 34|KIR genes, 28|Ras, 25|SH2, 22|CK, 21|reverse transcrip-tase, 21|ARF, 20|HLA, 20|HDL-C, 20|TNF</td><td>155|beta-catenin gene, 120|K-ras gene, 117|ras, 107|p53 gene, 97|ras gene, 95|K-ras mutation, 82|N-ras gene, 68|N-myc gene, 66|ras genes, 49|K-ras mutations</td><td>4|C2, 4|CAT, 3|ferritin, 3|C1, 3|growth factor, 3|insulin, 3|Ras, 3|SH2, 2|T1N1, 2|ras</td><td>45|NF-kappaB, 39|CD4, 34|IL-2, 32|NF-kappa B, 25|AP-1, 20|IL-4, 20|p65, 18|CD3, 18|CD8, 18|CD28</td></tr>
<tr><td></td><td>Wh7</td><td>304|kinase, 155|HLA, 128|Jun, 121|class I, 109|IFN, 86|growth factor, 81|antigen, 69|collagen, 67|TNF, 66|SH2</td><td>448|ras, 431|myc, 61|RAS, 53|fos, 39|N-myc oncogene, 38|Ha-ras, 37|kinase, 36|class I, 33|growth factor, 30|PrP</td><td>59|kinase, 26|Jun, 20|transcription fac-tor, 17|antigen, 17|SH2, 15|Sp1, 12|EGF, 12|growth factor, 12|binding protein, 12|fos</td><td>100|CD4, 99|IL-2, 62|NF-kappaB, 56|AP-1, 44|kinase, 37|Rel, 36|CD28, 34|p65, 29|IL-4, 27|CREB</td></tr>
<tr><td></td><td>SP</td><td>325|PCR, 152|a pro-tein, 134|cyclin, 128|Jun, 99|phosphatase, 98|ORF, 98|Ca2, 94|Ras, 92|IFN, 90|tumor necrosis factor</td><td>1552|ras, 471|myc, 359|PCR, 81|Ca2, 63|RAS, 62|AMP, 62|HCC, 56|fos, 56|cyclin, 55|GGT</td><td>33|cis, 26|Jun, 25|PCR, 21|a protein, 19|ORF, 18|cyclin, 17|SH2, 15|Sp1, 13|Asp, 13|EBP</td><td>100|CD4, 89|IL-2, 57|AP-1, 38|p65, 38|Rel, 37|CD28, 36|Th1, 31|EBP, 29|IL-4, 27|CREB</td></tr>
<tr><td></td><td>GP7</td><td>1234|has, 940|mRNA, 725|receptor, 569|in vitro, 460|enzyme, 431|RNA, 431|cDNA, 417|Escherichia coli, 393|receptors, 388|conserved</td><td>442|has, 406|mRNA, 360|PCR, 326|neuronal, 270|pancreatic, 232|receptor, 163|ras, 157|apoptosis, 152|oncogene, 140|cDNA</td><td>166|has, 95|cDNA, 75|serum, 74|RNA, 69|conserved, 67|mRNA, 61|in vitro, 60|plasma, 51|receptor, 46|kinase</td><td>213|lymphocytes, 190|receptor, 113|has, 106|mRNA, 105|in vitro, 98|IL-2, 97|CD4, 89|apoptosis, 77|receptors, 74|lymphoid</td></tr>
</table>

Table 11: **FP tagging errors (exact)** Similar to the previous table (cf. tbl. 10), this table shows the FP errors of the tagging solutions against the corpora. Again, exact matching has been used as evaluation measure.

<table>
<tr><th></th><th></th><th>FN - FsuPrge</th><th>FN - PennBio</th><th>FN - BC2</th><th>FN - Jnlpba</th></tr>
<tr><td>cos98</td><td>Ch2</td><td>278|cytokine, 242|KIR, 215|cytokines, 203|kinase, 176|transcription factor, 165|transcription factors, 75|RNA polymerase, 74|protease, 60|APP, 59|p53</td><td>385|ras, 329|p53, 277|polymerase, 266|beta-catenin, 204|K-ras, 148|MYCN, 115|c-kit, 55|KIT, 41|N-myc, 40|c-myc</td><td>8|neu, 5|LH, 5|chloramphenicol acetyltransferase, 4|CBF, 4|CREM, 4|CH, 4|prolactin, 3|D, 3|V, 3|S</td><td>39|cytokines, 27|transcription factors, 16|glucocorticoid receptor, 15|GR, 14|cytokine, 13|glucocorticoid receptors, 11|proteasome, 11|antibodies, 11|LTR, 10|Glucocorticoid receptors</td></tr>
<tr><td></td><td>Wh7</td><td>278|cytokine, 233|KIR, 215|cytokines, 141|kinase, 131|transcription factors, 119|transcription factor, 105|IL-1, 103|IL-12, 98|CD8, 94|HDL</td><td>404|N-ras, 403|N-myc, 273|polymerase, 157|c-kit, 136|MYCN, 81|VIP, 66|K-ras, 53|N-RAS, 51|p73, 50|KIT</td><td>20|CAT, 15|IgG, 11|IgM, 10|IgA, 9|neu, 9|PU, 9|NF-kappa B, 9|GH, 9|LH, 9|E1A</td><td>69|NF-kappa B, 46|GR, 39|cytokines, 35|Tax, 26|transcription factors, 17|IL-12, 15|nuclear factor-kappaB, 15|ER, 14|TCR, 14|cytokine</td></tr>
<tr><td></td><td>SP</td><td>278|cytokine, 215|cytokines, 203|kinase, 188|NF-kappaB, 176|transcription factor, 171|RNA polymerase, 165|transcription factors, 106|Tat, 105|IL-1, 103|IL-12</td><td>902|K-ras, 450|N-myc, 404|N-ras, 277|polymerase, 181|H-ras, 81|VIP, 63|Wnt, 53|N-RAS, 46|c-fos, 38|kinase</td><td>15|IgG, 11|IgM, 10|IgA, 9|PU, 9|NF-kappa B, 9|GH, 9|LH, 9|MAP kinase, 9|E1A, 9|NF-kappaB</td><td>86|NF-kappaB, 69|NF-kappa B, 46|GR, 39|cytokines, 35|Tax, 27|transcription factors, 17|IL-12, 15|nuclear factor-kappaB, 15|ER, 14|TCR</td></tr>
<tr><td></td><td>GP7</td><td>92|Tat, 70|Ig, 65|AR, 64|LDL, 47|sigmaK, 44|transcription factor, 40|SF-1, 39|sigma B, 38|gag, 36|sigma K</td><td>98|ras, 75|VIP, 69|p53, 28|T3, 24|telomerase, 21|N-MYC, 20|TH, 19|MK, 18|k-ras, 16|CRE</td><td>9|GH, 9|LH, 8|SH3, 6|Tat, 6|gag, 6|TCR, 5|NF-Y, 5|Rb, 4|Tax, 4|SH3 domains</td><td>46|GR, 35|Tax, 15|ER, 14|TCR, 12|Gadd45gamma, 11|antibodies, 11|LTR, 10|Cot kinase, 9|GRbeta, 9|IL-18Ralpha</td></tr>
</table>

Table 12: **FN tagging errors (cos98)** This table lists the FN errors for the tagging solutions against the gold standard corpora (based on cos98 matching).

<table>
<tr><th></th><th></th><th>FP - FsuPrge</th><th>FP - PennBio</th><th>FP - BC2</th><th>FP - Jnlpba</th></tr>
<tr><td>cos98</td><td>Ch2</td><td>35|p38, 28|Ras, 25|SH2, 22|CK, 21|reverse transcriptase, 20|HDL-C, 19|pp60, 18|HLA, 16|phox, 16|MHC</td><td>155|beta-catenin gene, 117|ras, 107|p53 gene, 97|ras gene, 66|ras genes, 43|ras oncogenes, 33|p53 protein, 27|c-kit gene, 26|p53 mutations, 24|ras oncogene</td><td>3|ferritin, 3|C1, 3|C2, 3|CAT, 3|growth factor, 3|insulin, 3|Ras, 2|T1N1, 2|ras, 2|OD1</td><td>38|CD4, 38|NF-kappaB, 32|IL-2, 23|NF-kappa B, 22|AP-1, 20|p65, 18|CD8, 16|p50, 16|TCR, 15|IL-4</td></tr>
<tr><td></td><td>Wh7</td><td>304|kinase, 153|HLA, 113|class I, 86|growth factor, 81|antigen, 69|collagen, 63|class II, 63|binding protein, 62|SH2, 59|Jun</td><td>448|ras, 431|myc, 60|RAS, 53|fos, 37|kinase, 33|growth factor, 29|antigen, 27|class I, 24|Myc, 23|MYC</td><td>59|kinase, 20|transcription factor, 17|antigen, 12|growth factor, 12|binding protein, 12|fos, 11|prolactin, 11|p53, 10|class I, 10|cyclin</td><td>87|CD4, 44|kinase, 42|NF-kappaB, 41|IL-2, 34|p65, 28|AP-1, 25|p50, 25|transcription factor, 24|IL-4, 23|CD28</td></tr>
<tr><td></td><td>SP</td><td>325|PCR, 152|a protein, 133|cyclin, 99|phosphatase, 98|ORF, 90|tumor necrosis factor, 89|cis, 88|Ca2, 86|Asp, 81|embryonic</td><td>1552|ras, 471|myc, 359|PCR, 80|Ca2, 62|RAS, 62|HCC, 56|fos, 56|cyclin, 55|cis, 54|GGT</td><td>33|cis, 25|PCR, 21|a protein, 18|cyclin, 17|ORF, 13|Asp, 13|protein kinase, 13|phosphatase, 12|EBP, 12|prolactin</td><td>87|CD4, 39|IL-2, 38|p65, 36|Th1, 31|EBP, 28|AP-1, 25|p50, 24|IL-4, 24|CTL, 23|CD28</td></tr>
<tr><td></td><td>GP7</td><td>1234|has, 940|mRNA, 725|receptor, 569|in vitro, 460|enzyme, 431|cDNA, 427|RNA, 417|Escherichia coli, 393|receptors, 388|conserved</td><td>442|has, 406|mRNA, 360|PCR, 326|neuronal, 270|pancreatic, 232|receptor, 163|ras, 157|apoptosis, 152|oncogene, 140|cDNA</td><td>166|has, 94|cDNA, 75|serum, 72|RNA, 69|conserved, 62|mRNA, 61|in vitro, 60|plasma, 51|receptor, 46|kinase</td><td>213|lymphocytes, 190|receptor, 113|has, 105|in vitro, 93|mRNA, 89|apoptosis, 87|CD4, 77|receptors, 74|lymphoid, 74|LPS</td></tr>
</table>

Table 13: **FP tagging errors (cos98)** The table gives an overview on the FP errors of the taggers (cos98 evaluation).
